# Supercomputer-Based Virtual Screening for Deoxyribonucleic Acid Methyltransferase 1 Inhibitors as Novel Anticancer Agents

**DOI:** 10.3390/ijms252211870

**Published:** 2024-11-05

**Authors:** Lara Johanna Friedrich, Axel Guthart, Min Zhou, Paola B. Arimondo, Thomas Efferth, Mona Dawood

**Affiliations:** 1 Department of Pharmaceutical Biology, Institute of Pharmaceutical and Biomedical Sciences, Johannes Gutenberg University, Staudinger Weg 5, 55128 Mainz, Germany; lfriedri@students.uni-mainz.de (L.J.F.); aguthart@students.uni-mainz.de (A.G.); minzhou1@uni-mainz.de (M.Z.); efferth@uni-mainz.de (T.E.); 2 Epigenetic Chemical Biology, Institute Pasteur, Université Paris Cité, CNRS UMR3523, 28 Rue du Docteur Roux, 75724 Paris, France; paola.arimondo@pasteur.fr

**Keywords:** cancer, DNA methylation, epigenetics, molecular docking and simulation, virtual drug screening

## Abstract

Targeting epigenetics is a new strategy to treat cancer and develop novel epigenetic drugs with anti-tumor activity. DNA methyltransferases transfer the methyl group from *S*-adenosyl-L-methionine (SAM) to the cytosine residue in a CpG island, leading to the transcription silencing of the gene. Hypermethylation can frequently be observed in several tumor types. Hence, the inhibition of DNMT1 has become a novel approach to cure cancer. In this study, virtual screening and molecular docking were performed for more than 11,000 ligands from the ZINC15 database to discover new hypomethylation agents. Four candidate compounds were further tested for their effects on DNMT1 in silico and in vitro. Compounds **2** and **4** showed the best DNMT1 inhibitory activity, but only compound **4** was able to inhibit the growth of several cancer cell lines. The hypomethylation of the luciferase gene by compound **4** was verified by a *CMV*- luciferase assay using KG-1 cells. Additionally, compound **4** suppressed cell migration in a dose- and time-dependent manner in the wound healing assay. Moreover, cell cycle analyses demonstrated that compound **4** arrested CCRF-CEM cells and MDA-MB-468 cells in the G0/G1 phase. Also, compound **4** significantly induced early and late apoptosis in a dose-dependent manner. In conclusion, we introduce compound **4** as a novel DNMT1 inhibitor with anticancer activity.

## 1. Introduction

Genetic and epigenetic modifications play a fundamental role in cancer initiation and progression. Conrad Waddington first established the term epigenetics to describe the mechanisms altering gene expression without changing the DNA sequence [1]. Epigenetic processes are heritable and reversible and include a multitude of known processes. These include DNA methylation, histone modification, chromatin remodeling, and non-coding RNA-induced modification, all of which are critical mechanisms in the physiological growth and development of cells. However, upon deregulation, they contribute to pathological development such as carcinogenesis [2].

DNA methylation is a process occurring across all major groups of living organisms, including plants, animals, fungi, and bacteria; however, it does not always serve the same purposes. In prokaryotes, methylation targets the DNA base adenine and cytosine and contributes to hindering phage attacks, as well as chromosome replication and repair [3,4,5]. In eukaryotes, the methylation of the DNA base cytosine plays a major role in gene expression, embryonic development, X-chromosome inactivation, and carcinogenesis [6].

DNA methylation is the transfer of a methyl group from *S*-adenosyl-L-methionine (SAM) to the carbon atom on position 5 of a cytosine residue in a CpG dinucleotide, forming 5-methylcytosine [7]. This process is catalyzed by a family of DNA methyltransferases (DNMTs) consisting of DNMT1 and DNMT3. DNMT1 is a large protein with multiple domains, which primarily targets hemi-methylated DNA and is therefore strongly associated with methylation after replication as opposed to de novo methylation, which is catalyzed mainly by DNMT3A and 3B [8]. In normal cells, DNA methylation and demethylation are tightly regulated. However, in cancer cells, this balance is disrupted, and a change in DNA methylation patterns can be observed. This bimodal deregulation plays a role in carcinogenesis, as hypermethylation may lead to the inactivation of tumor suppressor genes, while hypomethylation has been associated with the upregulation of oncogenes [9]. As the abnormal activity of DNMTs in DNA methylation plays a significant role in carcinogenesis, DNMT inhibition represents a potential target for novel cancer therapy [10].

Worldwide, nearly 70 DNMT inhibitors (DNMTi) are being investigated. As a proof of principle, currently, two DNMTis, azacitidine and decitabine, have been approved by the FDA for treating acute myeloid leukemia (AML), as well as myelodysplastic syndrome (MDS) [11]. A clinical trial for azacytidine, which is the first DNMTi in clinical practice, demonstrated that after the subcutaneous administration of 75 mg/m^2^ azacytidine for seven consecutive days every four weeks, the overall patient response was 13.9%. Notably, a favorable bioavailability of 89% after injection was observed [12]. Interestingly, leading to its FDA approval in 2006, decitabine showed 30 times more inhibitory activity toward DNMT in patients than azacytidine [12]. As cancer patients are often faced with serious side effects from traditional anticancer treatments, such as cytotoxic chemotherapy, there is a continuous need for the development of novel anticancer treatments. Therefore, DNA methyltransferases, specifically DNMT1, are an important target for the development of novel anticancer drugs.

The present investigation aimed to identify new DNMT1 inhibitors with anticancer activity. Therefore, more than 11,000 compounds were downloaded from the ZINC15 database for in silico studies. After virtual screening and molecular docking against DNMT1, four candidate compounds were selected for molecular dynamic simulation and in vitro testing as DNMT1 inhibitors. Furthermore, the ability of the identified compound **4** to demethylate the cytomegalovirus (CMV) promoter in a stable cellular luciferase–CMV reporter system was investigated. The effects of compound **4** on cellular processes, such as cell migration, cell cycle, and apoptosis, were also studied.

## 2. Results

### 2.1. Virtual Drug Screening and Molecular Docking

**Figure 1:**
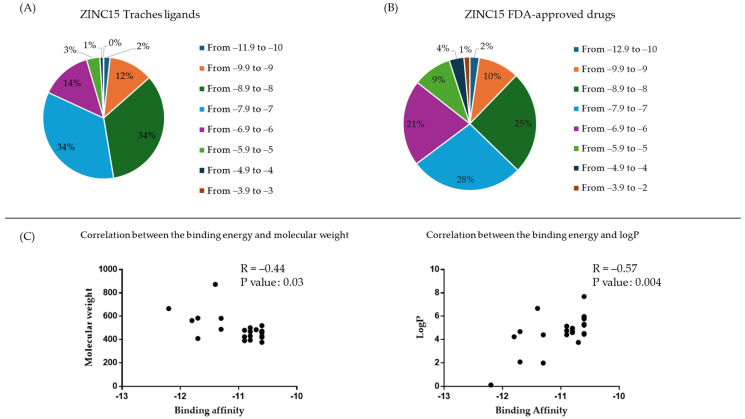
(**A**,**B**) Pie chart showing the percentage of ligands from ZINC15 tranches and ZINC15 FDA-approved drugs belonging to the category of lowest binding energy (kcal/mol) ligands screened using PyRx software. (**C**) Correlation coefficient of lowest binding energy (kcal/mol) vs. molecular weight, as well as lowest binding energy and logP.

Virtual screening was used to assess the binding affinity of more than 11,000 compounds to DNMT1. These compounds were selected from two subsets of the ZINC15 database and the FDA-approved subset, and the three-dimensional structures were downloaded from https://zinc15.docking.org, accessed on 7 November 2022. Virtual screening using PyRx software version 1.5.6 estimated the binding affinities of ≤−9 kcal/mol of 13% and 12.2% of the ZINC15 subset and FDA compounds, respectively (Figure 1). Correlation analyses were carried out between the molecular weight and logP of the ligands and the predicted binding affinities. As shown in Figure 1, there was no significant correlation between the molecular weights and the binding energies, while logP showed a slight correlation, with an *r*-value of 0.57 and a *p*-value of 0.004.

Aiming to confirm the obtained results, molecular docking using AutoDock 2.4.6 was performed on the top 22 compounds based on PyRx-based virtual screening (Table 1).

**Figure 2:**
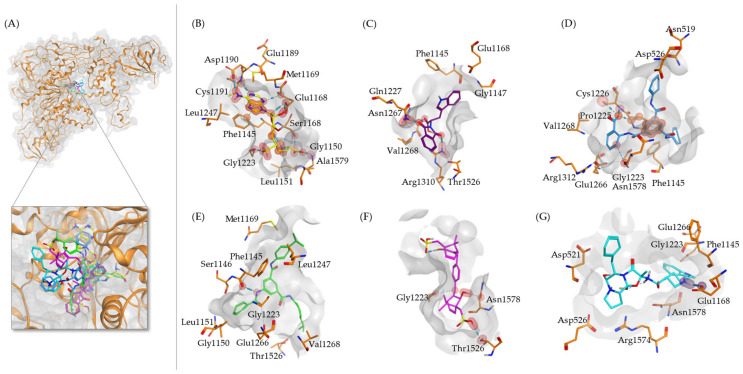
Molecular docking of DNMT1 (PDB ID: 4WXX) with candidate inhibitors using AutoDock 4.2.6. (**A**) The complete structure of DNMT1 (orange) is shown along with the best poses of all docked ligands and a zoomed illustration into the SAH binding pocket. (**B**–**G**) The pose for SAM (yellow) and the best docking poses of known inhibitor RG108 (purple) and compounds **1** (light blue), **2** (green), **3** (magenta), and **4** (cyan) are shown. Each panel includes the 3D pose in the binding pocket (gray surface) and residues within 4 Å that are interacting with the compounds. Hydrogen bonds are depicted by red spheres, and orange spheres show interactions with the π-system of aromatic rings. The interacting atoms are connected by dashed lines.

Using the Lamarckian algorithm, we were able to reveal the compounds with the lowest binding energies, the predicted inhibition constant, and the amino acids involved in the interactions. These findings are summarized in Table 2. Figure 2 illustrates the interactions among the top four selected compounds with the lowest binding affinities to DNMT1.

Of all compounds shown in Table 2, the top four were selected as candidates for further investigation. In silico methods might not necessarily reflect the full reality, but they are valuable in offering initial insights into molecular interactions. To better understand the in silico data obtained from AutoDock studies, the top four compounds were selected for further docking in MOE, focusing on the entire DNTM1 binding site. The docking simulation results are presented in Table 3, which includes docking scores and the interacting amino acids for the best docking pose of each compound.

**Figure 3:**
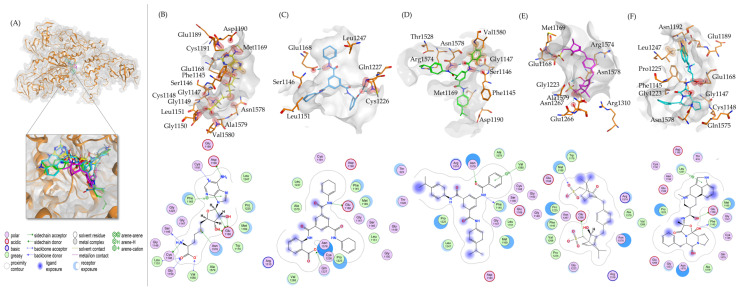
Molecular docking of DNMT1 (PDB ID: 4WXX) with candidate inhibitors using MOE. (**A**) The complete structure of DNMT1 (orange) is shown, along with the best poses of all docked ligands and SAH in the SAH binding site. (**B**) The pose of SAH (yellow) is displayed, illustrating its 3D position within the binding pocket and the corresponding 2D interaction map. (**C**–**F**) The best docking poses for compounds **1** (light blue), **2** (green), **3** (magenta), and **4** (cyan) are shown. Each panel includes the 3D pose in the binding pocket (gray surface) and a 2D interaction map. Residues within 4 Å that are in contact with the compound are shown in orange. Hydrogen bonds are depicted by red spheres, and orange spheres show interactions with the π-system of aromatic rings. The interacting atoms are connected by dashed lines.

For more detailed insights, Figure 3 provides a 3D representation of the DNMT1 binding pocket, featuring a 2D interaction map with various types of interactions between the compounds and DNMT1. The most robust interactions were obtained for compound **1**, particularly forming three hydrogen bonds with the residues Glu1168, Cys1226, and Gln1227. This resulted in the most favorable docking score among the tested compounds, with a score of −10.03 ± 0.16 kcal/mol. Notably, Cys1226 serves as the catalytic cysteine in DNMT1 and plays an important role in the enzyme’s mechanism by forming a transient covalent bond with the DNA during the methylation process [13,14]. Compound **3** exhibited a higher docking score (−8.08 ± 0.29 kcal/mol), indicating less affinity to DNMT1. The optimal binding pose of this compound formed hydrogen bonds with different residues than those found for the control ligand SAH, indicating less favorable binding interactions (Table 3). Considering the standard deviation, compounds **4** and **2** also exhibited favorable docking scores of −9.85 ± 0.14 kcal/mol and −9.76 ± 0.15 kcal/mol, respectively, indicating comparable performances to compound **1**. If comparing the hydrogen-bond-forming amino acids of DNMT1 with SAH in the used crystallographic structure (Table 3), the docking simulation results reveal that Glu1168 stands out as a common interacting residue. Hydrogen bonds with this residue were observed in the best poses of the compounds **1**, **2**, and **4**. Interactions with the catalytic Cys1226 could be observed in the best poses of compounds **1** and **4**.

In the crystal structure of the DNMT1-SAH complex, it was observed that 21 amino acids were interacting. Upon comparing these amino acids with those found in the docking studies, it was determined that compound **1** interacts with 19 of the 21 amino acids (90%), while compounds **2** and **4** each interact with 17 amino acids (81%). In contrast, compound **3** interacts with only 10 amino acids (48%). Compounds **1**, **2**, and **4** demonstrate a high similarity in interacting amino acids compared to those of SAH in the DNMT1 binding pocket, whereas the similarity is lower for compound **3**. This is consistent with the lower docking scores for compound **3** (Table 3).

### 2.2. Molecular Dynamics Simulations for Protein–Ligand Complexes

**Figure 4:**
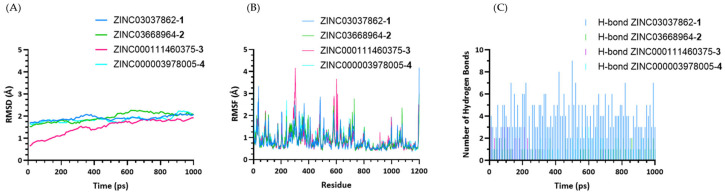
Molecular dynamics (MD) simulations. (**A**) RMSD plots of the DNMT1–compound complexes during 1000 ps of MD simulation time. (**B**) RMSF values in correlation to specific residues. (**C**) Hydrogen bond analysis with a cutoff of 3.5 Å during MD simulations. In all plots, complexes of DNMT1 with compounds **1** (blue), **2** (green), **3** (magenta), and **4** (cyan) are shown.

Molecular dynamics (MD) simulations were performed to evaluate the stability and flexibility profiles of the compounds in the DNMT1 binding site. Short videos of these MD simulations can be found in the Supplementary Materials S1–S4. The RMSD values of the DNMT1–compound complexes relative to the initial docked structure obtained from MOE molecular docking are shown in Figure 4.

The DNMT1–compound **4** complex displays RMSD values ranging from 1.67 Å to 2.23 Å, with an average of 1.86 Å. These results indicate relatively stable binding with moderate fluctuations. Complexes of compounds **1** and **2** showed similar stability profiles, with RMSD values ranging from 1.69 Å to 2.26 Å for compound **1** and from 1.53 Å to 2.26 Å for compound **2**. These compounds maintain stable interactions. In contrast, the DNMT1–compound **3** complex showed RMSD values ranging from 0.64 Å to 2.26 Å, with an average of 1.42 Å, which indicates stability but with a broader range of fluctuation compared to the other evaluated complexes. The RMSF values for the residues of DNMT1, which indicate the flexibility and movement of specific regions, are shown in Figure 4B. In the DNMT1–compound **4** complex, the RMSF values fluctuated between 0.36 Å and 3.34 Å, indicating that this complex exhibits the most minor fluctuation and most stable interactions among the tested compounds. The DNMT1–compound **1** complex displays the highest range of RMSF values ranging from 0.38 Å to 4.17 Å, showing moderate flexibility. Similarly, the DNMT1–compound **3** complex exhibited RMSF values ranging from 0.38 Å to 4.16 Å. The DNMT1–compound **2** complex exhibited RMSF values from 0.38 Å to 2.98 Å, reflecting a stable interaction with moderate flexibility. To investigate the stability of hydrogen bonds within DNTM1–compound complexes, the number of hydrogen bonds during MD simulations with a cutoff of 3.5 Å was calculated (Figure 4C). The complexes DNMT1–compound **3** and DNMT1–compound **4** showed similar ranges of hydrogen bonds, fluctuating between 0 to 3, and for the complex DNMT1–compound **2**, the range is between 0 and 2. This indicates that these complexes have moderate interaction stability. However, it is important to note that the DNMT1–compound **3** complex frequently did not form any hydrogen bonds. This indicates the least stable interaction in terms of hydrogen bond formation among the tested compounds. In contrast, the results of the DNMT1–compound **1** complex show a broader range of 1 to 9 hydrogen bonds, suggesting its capability to form a high number of hydrogen bonds and indicating significant flexibility in its binding.

### 2.3. Compounds ***2*** and ***4*** Inhibit DNMT1 Activity

**Figure 5:**
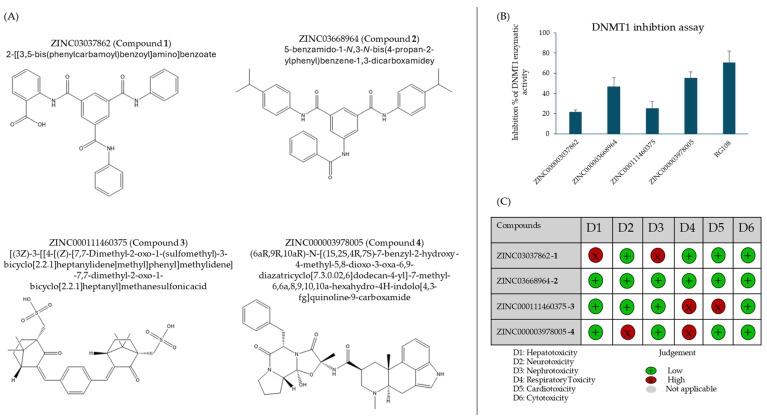
(**A**) Chemical structures of compounds **1**, **2**, **3,** and **4** and (**B**) DNMT1 inhibition assay. Data are represented as mean ± SD of three independent experiments. (**C**) Predicted toxicities of selected compounds based on structural similarity to compounds known to evoke toxicity in selected physiological functions.

As a next step, the influence of the top four compounds revealed by virtual screening and molecular docking on DNMT1 in vitro activity was investigated. Two compounds were from the ZINC15 subset, and two were from the FDA-approved drug library. Figure 5 showed that compounds **2** and **4** exhibited significant inhibitory activity at 10 µM of 46.4% and 55.3%, respectively. These results underline the previously in silico-determined strong binding affinity of compounds **2** and **4** to DNMT1. The known DNMT1 inhibitor RG108 was used as a positive control and displayed an inhibition value of 70.5%. Based on these inhibition results, compounds **2** and **4** were considered for further analysis.

### 2.4. Cytotoxic Effects of Potential DNMT1 Inhibitors

**Figure 6:**
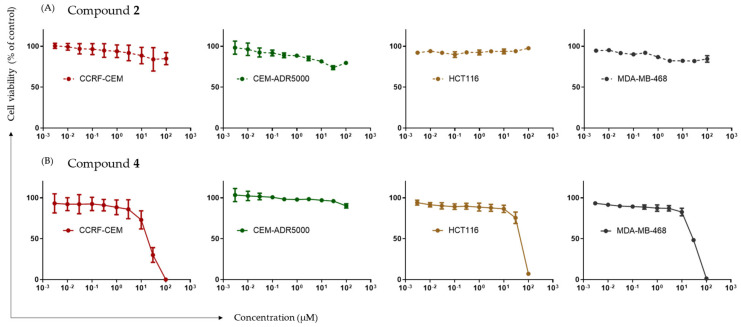
Cytotoxic effects of compounds **2** and **4** as determined by resazurin reduction assay. Dose–response curves of compound **2** (**A**) and compound **4** (**B**) obtained with sensitive CCRF-CEM and multidrug-resistant P-glycoprotein overexpressing CEM-ADR5000 leukemia cells, as well as HCT116 colon cancer and MDA-MB-468 breast cancer cell lines after treatment with compounds for 72 h. Data shown resulted from mean values and standard deviation values obtained from three independent experiments.

Based on molecular docking in silico and DNMT1 activity inhibition in vitro, the compound selection was narrowed down to compounds **2** and **4,** which were then investigated for cytotoxicity by means of a growth inhibition assay. Using the resazurin reduction method, cytotoxicity was examined toward CCRF-CEM and CEM/ADR5000 leukemia cells, HCT116 colon carcinoma cells, and MDA-MB-468 breast cancer cells after 72 h of treatment. As visualized in the dose–response curves of the two selected drug candidates in the four cancer cell lines, only compound **4** displayed significant cytotoxic effects (Figure 6).

In contrast, compound **2** did not exert significant cytotoxicity in any of the selected cell lines. Table 4 reveals that compound **4** had the strongest cytotoxic effects against the sensitive leukemia cell line CCRF-CEM, with an IC_50_ value of 18.25 ± 4.37 µM. Significant cytotoxic effects can also be seen toward HCT116 cells (IC_50_ value of 46.82 ± 3.04 µM), as well as MDA-MB-468 cells (IC_50_ value of 29.42 ± 2.37 µM). This underlines the ability of compound **4** to inhibit the growth of hematopoietic and solid tumors.

Cytotoxicity toward the multidrug-resistant leukemia subline CEM/ADR5000 was not observed for either compound, even at concentrations up to 100 µM, indicating that CEM/ADR5000 cells were cross-resistant to these compounds.

As compound **4** displayed a promising binding affinity in silico, as well as exerting the strongest DNMT1 activity inhibition and cytotoxic effects, additional analyses were only performed on this compound.

### 2.5. Compound ***4*** Demethylates the Promotor of the CMV-Luciferase Gene Construct

**Figure 7:**
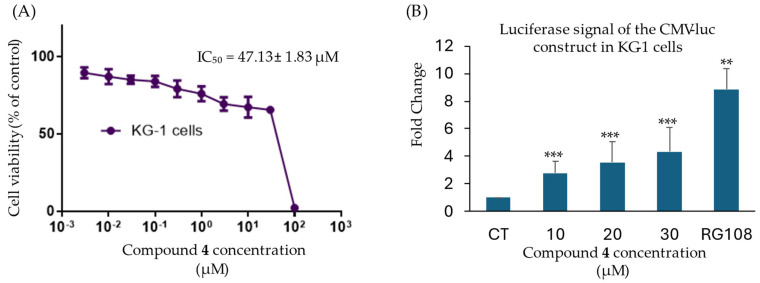
(**A**) Cytotoxicity dose–response curves of compound **4** on KG-1 cells determined by the resazurin reduction assay. (**B**) Dose-dependent fold induction of CMV-luc luciferase signal after KG-1 cells were treated with compound **4** compared to the negative control, DMSO, and the positive control compound, RG108. (** *p* < 0.01; *** *p* < 0.001).

KG-1 CMV-luc leukemia cells were treated with compound **4** to study its ability to demethylate CMV promoter via the inhibition of DNMT1 and reactivate the luciferase reporter gene. Interestingly, compound **4** induces the luciferase signal with a 2.7-fold change at 10 μM after 24 h of treatment (Figure 7). The luciferase fold change was significantly increased by compound **4** in a dose-dependent manner, providing strong evidence supporting the hypomethylating properties of compound **4**. Since the KG-1-CMV-luc cells are highly sensitive to treatment with suitable ligands, the cytotoxicity of compound **4** toward KG-1 CMV-luc leukemia cells was determined using the resazurin assay. As shown in Figure 7, the IC_50_ value of compound **4** was 47.13 ± 1.83 µM, which was higher than the concentration used for the CMV-luciferase assay.

### 2.6. Compound ***4*** Inhibited 2D Migration of MDA-MB-468 Cells and HCT116 Cells

**Figure 8:**
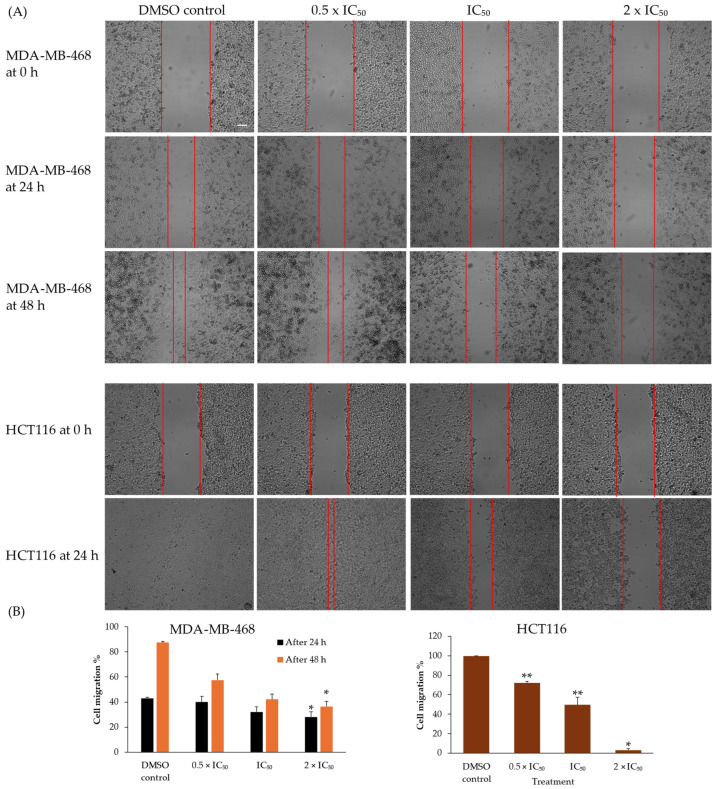
Compound **4** inhibited cell migration of MDA-MB-468 and HCT116 cells in a dose-dependent manner. (**A**) Representative photographs of MDA-MB-468 and HCT116 cells showing cell migration 24 h and 48 h after treatment with 0.5 × IC_50_, IC_50_, and 2 × IC_50_ of compound **4** or DMSO. Cell migration pictures were taken with 1× magnification. Scale bar 100 µM: (**B**) Quantification of migration area and cell invasion calculated using ImageJ and shown as mean values ± SD. (* *p* < 0.05; ** *p* < 0.01 vs. DMSO control).

As cell migration plays an essential role in tumorigenesis [15], the inhibitory properties of compound **4** on MDA-MB-468 breast cancer cells and HCT116 colon cancer cells were investigated in vitro. A wound healing assay was used to determine cell migration after treatment with varying concentrations of compound **4** compared to control cells treated with DMSO. A concentration-dependent decrease in cell migration and wound healing was observed in both cell lines with increasing doses of compound **4** (Figure 8). After 24 h, wound healing was quantified in MDA-MB-468 cells as 43.1%, 40%, 32.1%, and 28.2% and in HCT116 cells as 99.8%, 72.2%, 49.5%, and 3.1% after treatment with DMSO at doses of 0.5 × IC_50_, 1 × IC_50_, and 2 × IC_50_ of compound **4**, respectively. After 24 h, the wound of the MDA-MB-468 control group was not fully healed, leading us to continue incubation for another 24 h. After 48 h, the wound healing rates of MDA-MB-468 cells were 87.6%, 57.6%, 42.1%, and 36.5%, after treatment with DMSO at doses of 0.5 × IC_50_, 1 × IC_50,_ and 2 × IC_50_ of compound **4**, respectively. It is evident that 48 h after treatment, two doses of compound **4** treatment led to a significant decrease (*p* < 0.003) in the cell migration of breast cancer cells, and colon cancer cells. We were therefore able to elucidate the in vitro time- and concentration-dependent inhibitory effect of compound **4** on breast cancer and colon cancer cell migration.

### 2.7. Effect of Compound ***4*** on the Cell Cycle and KI-67 Expression

**Figure 9:**
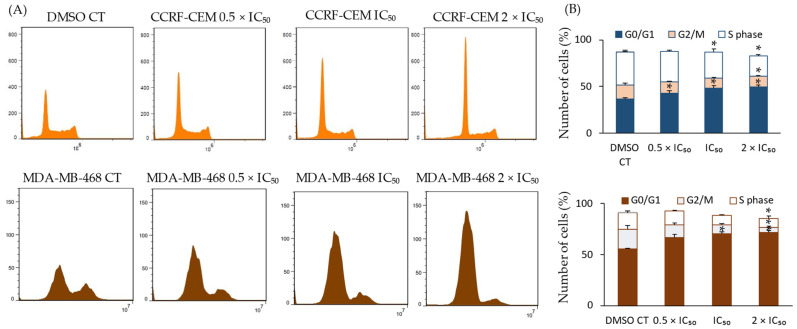
Cell cycle arrest of CCRF-CEM and MDA-MB-468 cells in the G0/G1 phase induced by compound **4**. (**A**) different cell cycle phases as represented by flow cytometry. (**B**) Percentages of cells in separate cell cycle phases post-treatment with 0.5 × IC_50_, IC_50_, and 2 × IC_50_ of compound **4** are shown. A dose-dependent induction of cell cycle arrest in the G0/G1 phase was observed, accompanied by a decrease in the cell populations in the S-phase and G2/M phases. (* *p* < 0.05 vs. DMSO control).

In order to better elucidate the inhibitory effects that compound **4** exerted toward CCRF-CEM and MDA-MB-468 cells, the cell cycle distribution of CCRF-CEM and MDA-MB-468 cells after treatment with varying concentrations of compound **4** was analyzed in comparison to treatment with the negative control, DMSO. After treatment with DMSO or 0.5 × IC_50_, 1 × IC_50_, or 2 × IC_50_ of compound **4**, the percentages of the CCRF-CEM population in the G0/G1 phase increased from 36.6% to 42.8%, 48.2%, and 49.2%, respectively, and those of the MDA-MB-468 population in the G0/G1 phase increased from 55.2% to 66.4%, 70.1%, and 71.3%, respectively, as presented in Figure 9. A slight dose-dependent decrease in CCRF-CEM cell populations in the G2/M phase, namely, 15.3%, 12.48%, 10.8%, and 11.8%, respectively, could also be observed. Similarly, in MDA-MB-468 cells, a slight dose-dependent decrease in populations in the G2/M phase, namely, 19.3%, 12.8%, 8.8%, and 5.1%, respectively, could also be observed. The percentages of the CCRF-CEM cell population in the S-phase decreased from 35.1% to 32.6%, 28%, and 21.9%, respectively. In MDA-MB-468 cells, the percentages of the population in the S-phase also decreased from 16.1% to 13.3%, 9.4%, and 9.1%, respectively. It can be summarized that compound **4** induced dose-dependent G0/G1 phase arrest and, correspondingly, a decrease in the G2/M and S phases in both cell lines compared to the untreated control cells.

**Figure 10:**
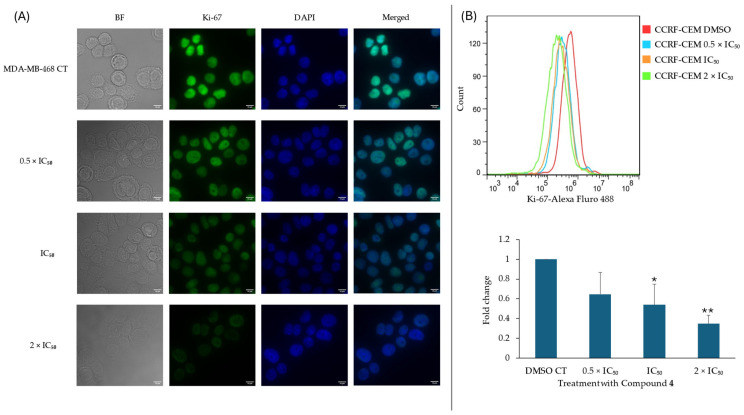
The expression of Ki-67. (**A**) Immunofluorescence of Ki-67 in MDA-MB-468 treated with three concentrations of compound **4** for 24 h. Cells images stained with DAPI (blue). Scale bars: 10 μM. (**B**) Fluorescence expression of Ki-67-Alexa Fluro 488 in CCRF-CEM cells. Flow cytometry showed a reduction in Ki-67 green fluorescence after compound **4** treatment. Bar chart represents the mean ± SD of two independent experiments (* *p* < 0.05; ** *p* < 0.01).

To further underline cell cycle results, an immunofluorescence analysis using MDA-MB-468 cells and Ki-67 was conducted. Post-treatment with DMSO or 0.5 × IC_50_, 1 × IC_50_, or 2 × IC_50_ of compound **4**, the localization and expression of Ki-67 within the cell was investigated, using DAPI staining as a point of reference. It can be seen in Figure 10 that Ki-67 is a nuclear protein that decreases in a dose-dependent manner after 24 h of incubation with compound **4**. As shown in Figure 10, flow cytometry was performed using CCRF-CEM cells to examine expression change in the proliferation marker Ki-67 after treatment with compound **4**. This elucidated a dose-dependent decrease in the Ki-67 fold-change after treatment with compound **4**. A fold change of 1 for the CT group treated with DMSO leads to fold changes of 0.64, 0.54, and 0.35 after treatment with 0.5 × IC_50_, 1 × IC_50_, or 2 × IC_50_ of compound **4**, respectively.

### 2.8. Compound ***4***-Induced Late Apoptosis of CCRF-CEM Cells via Deregulation of Apoptosis Markers

**Figure 11:**
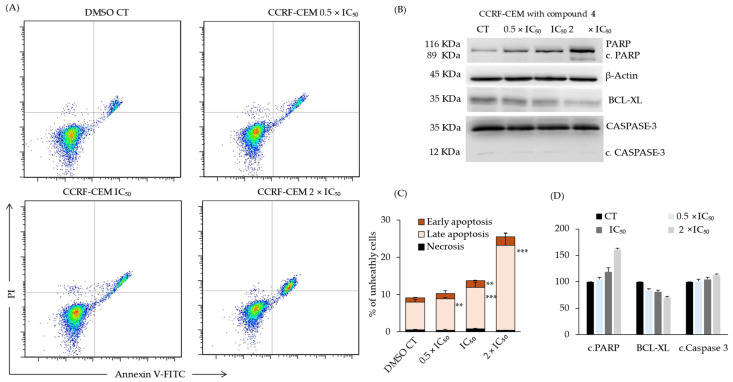
Cell death detection in sensitive CCRF-CEM cells using annexin V/PI staining and western blot. (**A**) Cells were treated with 0.5 × IC_50_, IC_50_, and 2 × IC_50_ of compound **4** or DMSO, and results were obtained using flow cytometry. Viable cells are characterized by annexin V (−)/PI (–), early apoptotic cells are characterized by annexin V (+)/PI (–), late apoptotic/necrotic cells are characterized by annexin V (+)/PI (+), and dead cells are characterized by annexin V (–)/PI (+). (**B**) Western blot analysis of apoptosis marker PARP, BCL-XL, and caspase-3 in CCRF-CEM cells treated with compound **4** (0.5 × IC_50_, IC_50_, and 2 × IC_50_) for 24 h. (**C**,**D**) Quantification results for flow cytometry and western blot. (** *p* < 0.01, *** *p* < 0.001 vs. DMSO control).

DNMT1 has the ability to modulate crucial genes and pathways involved in cell survival [16]. Thus, the influence of compound **4** on the apoptotic behavior of leukemia cells was evaluated. The flow cytometry results in Figure 11 revealed that CCRF-CEM cells treated for 24 h with increasing concentrations of compound **4** display significantly higher levels of apoptosis than cells treated with the negative control, DMSO. Viable cells that are dual negative for PI and annexin V can be found in quadrant Q1, with necrotic cells binding high levels of PI but low levels of annexin V in Q2, late apoptotic cells binding high levels of both PI and annexin V in Q3, and early apoptotic cells binding low levels of PI and high levels of annexin V in Q4. Predominantly, late apoptosis was triggered by compound **4** in a concentration-dependent manner, ranging from 7.5% in cells treated with DMSO to 22.8% in cells treated with 2 × IC_50_ of compound **4** (Figure 11). A slight concentration-dependent increase in early apoptosis could be observed from 1.1% in cells treated with DMSO to 2.3% in cells treated with 2 × IC_50_ of compound **4**. Thus, compound **4** was able to significantly augment the induction of late apoptosis in CCRF-CEM cells in a dose-dependent manner. To further analyze compound **4**-induced apoptosis, a western blot was conducted to reveal the effect of compound **4** on pro- and anti-apoptotic protein expression in CCRF-CEM cells. As can be seen in Figure 11, compound **4** causes a dose-dependent upregulated expression of the proapoptotic proteins PARP and cleaved PARP, ß-actin, and CASPASE-3 and a downregulated expression of the anti-apoptotic protein BCL-XL (Supplementary Materials S5–S9).

## 3. Discussion

Dysregulation of the epigenetic landscape is a characteristic feature often associated with a plethora of diseases, including cancer. A crucial epigenetic modification in cancer development and progression is DNA methylation, which is modulated by the methyltransferase DNMT1. Among the multitude of effects methylation has on cancer development, the hypermethylation of gene promoters, such as tumor suppressor genes, plays an important role in the carcinogenesis of liver cancer, breast cancer, and many other tumor types. As methylation leads to gene inactivation, tumor suppressor genes, such as *WT1*, *TP53*, and *MADR2*, lose their function, resulting in interference in the regulation of pathways controlling DNA repair, apoptosis, and tumor proliferation. The importance of the maintenance of such genes is highlighted by the discovery that *TP53* mutations could be identified in up to 50% of all cancers [17]. In addition to the genetic mutations, the silencing of *TP53* by epigenetic mechanisms has been reported [18]. At the moment, the only two DNMT1 inhibitors approved by the FDA are azacitidine and decitabine for the treatment of acute myeloid leukemia and myelodysplastic syndrome [19]. However, as they are nucleoside analogs and are integrated into DNA and RNA, they may instigate serious side effects [20]. Another DNMT1-selective inhibitor (GSK3685032) was developed with transcriptional activation and cancer cell growth inhibition properties. GSK3685032 binds to the DNA-protein interface of DNMT1, interfering with the interaction between DNMT1 and DNA [21]. Investigating methylation patterns as biomarkers in clinical diagnostics is also of increasing significance. Methylation profiles help differentiate between healthy tissue and cancer cells and play a role in identifying different types of cancer. Using methylation array technology, the methylation status of 96% of CpG islands of the human genome may be investigated and therefore plays a role in many diagnostic processes, such as identifying the primary tumor of metastasis, as well as being important for the choice of further treatment of the tumor [22]. As cancer remains the second leading cause of death worldwide, and chemotherapy is commonly applied, despite a vast number of serious side effects, novel therapeutic possibilities, such as DNMT1 inhibitors, must be further explored. The unmet therapeutic needs in cancer treatment, as well as the scarce amount of DNMT1 inhibitors on the market, prompted us to further investigate the development of novel non-nucleotide DNMT1 inhibitors.

In this study, we examined a selection of potential DNMT1 inhibitors and were able to report that the ergotamine derivative compound **4** exerted significant inhibitory effects on DNMT1 in silico and in vitro and indeed exhibited a plethora of anticancer effects. To begin with, a library of compounds potentially binding to DNMT1 was constructed and screened in silico toward DNMT1 to examine the binding affinities between the drug and target protein. Interestingly, binding interaction visualization of our top four compounds revealed specific interactions with key residues, such as Glu1168 and Cys1226. These compounds bind within similar regions of DNMT1, particularly the SAM-binding pocket, which suggests that they may share similar mechanisms of DNMT1 inhibition. However, the demethylated metabolite *S*-adenosyl-L-homocysteine (SAH) is also capable of binding to DNMT1, thereby inhibiting its ability to catalyze a methyl transfer to DNA [23]. Upon analyzing the binding properties of SAH to DNMT1, Glu1168 was among the interacting residues. Interestingly, Glu1168 was also an interacting residue in compounds **1**, **2**, **3**, and **4**, revealing a similar binding mode as the known DNMT1 ligand SAH and validating these compounds as possible novel DNMT1 inhibitors.

As a next step, the in vitro inhibitory effect of the top four selected compounds (**1**, **2**, **3**, and **4**) was investigated. A DNMT1 activity inhibition assay revealed that compound **2** and compound **4** significantly inhibited the enzymatic function of DNMT1, exhibiting an inhibition at 10 µM of 46.4% and 55.3%, respectively. Interestingly, compounds **1** and **3** showed only low levels of DNMT1 inhibition at 21.6% and 25.1%, respectively, leading to their exclusion in further investigations. Compounds **2** and **4**, therefore, progressed onto cytotoxicity testing in four cancer cell lines. When developing novel anticancer agents, it is crucial that these agents display cytostatic effects by inhibiting factors that are otherwise advantageous to cancer cells [24]. In this case, the cytotoxicity linked to DNMT1 inhibition was experimentally demonstrated by the resazurin reduction assay. Compound **4** exerted significant anticancer effects on the cancer cell lines CCRF-CEM, HCT116, and MDA-MB-468. Notably, compound **2** did not show considerable cytotoxicity toward any selected cell lines. Cancer cells are normally characterized by their ability to proliferate at high rates. One explanation is the silencing of tumor suppressor genes by mechanisms such as hypermethylation catalyzed by DNMT1. Thus, the inhibition of DNMT1 through compound **4** and the subsequent demethylation and activation of tumor suppressor genes may explain the cytotoxic effects observed. Furthermore, DNMT inhibitors promoted NK cell-mediated cytotoxicity in tumors, which should be considered an additional mechanism of action.

Following previous investigations, compound **4** was chosen as the top DNMT1 candidate inhibitor of interest for further evaluation. Compound **4** represents the FDA-approved drug dihydroergotamine belonging to the class of ergot alkaloids used to treat headaches and migraines by triggering the vasoconstriction of intracranial blood vessels [25]. In the past years, the drug repurposing concept has experienced a significant increase in attention since many pre-clinical efforts and financial investments ought to be saved, leading to a highly efficient identification of new therapeutical possibilities [26].

As the presence of tumor metastasis is closely linked to poor survival prognosis, the link between cell migration and compound **4** was analyzed. We found that compound **4** visibly inhibited MDA-MB-468 and HCT116 cancer cell migration in a dose- and time-dependent manner. A hallmark of malignant cell invasion is the epithelial–mesenchymal transition (EMT), in which epithelial cells transition to a mesenchymal phenotype, characterized, for example, by the upregulation of vimentin and fibronectin [27]. Among others, EMT processes are modulated by the WNT signaling pathway, which in turn is modulated by methylation patterns and can therefore be influenced by DNMT1 regulation. As previously reported, metastatic cancer is responsible for 90% of cancer deaths; any potential anticancer drug might also have to limit cell migration and metastasis, which supports the results observed in this study.

As postulated by Hanahan and Weinberg, one of the six main hallmarks of cancer includes the ability to resist cell death [28]. The apoptotic trigger is regulated by several upstream and downstream signaling factors, including the BCL-2 protein family. Within this protein family, the pro-apoptotic effectors BAX and BAK have the ability to destroy the outer mitochondrial membrane, triggering other proapoptotic proteins to be released and setting off a proteolytic cascade that ultimately leads to the apoptosis of the cell [29]. An increase in pro-apoptotic BCL-2 proteins after a DNMT1 blockade [29] could restore cell death, which tumor cells would otherwise avoid. This evidence prompted us to investigate the induction of apoptosis via DNMT1 inhibition in CCRF-CEM leukemia cells. Indeed, a significantly increased induction of late apoptosis ranging from 7.5% in untreated cells to 22.8% in cells treated with 2 × IC_50_ of compound **4** was confirmed. Interestingly, another study found that the overexpression of DNMT1 not only affected apoptosis but also played a significant role in evading apoptosis in cardiomyocytes [30]. A western blot analysis revealed that the known proapoptotic proteins, ß-actin, PARP, and CASPASE-3, were dose-dependently upregulated after treatment with compound **4**, which is consistent with the observation of increased apoptosis. ß-Actin is a vital part of the cytoskeleton that modulates a plethora of essential cellular processes, including migration, cell division, and gene expression regulation [31]. In accordance with this, studies have found that agents increasing ß-actin prevalence can be considered useful anticancer agents [32] Furthermore, a larger population of cells in the G0/G1 cell cycle phase were arrested upon treatment, as opposed to in untreated control cells. These cell populations were apparently unable to progress to the DNA synthesis phase of replication, ultimately leading to reduced proliferation and quiescence. These findings are in line with our results, indicating that compound **4** dose-dependently increased apoptosis and thereby decreased tumor cell proliferation. Through further examination, it was detected that compound **4** induced a dose-dependent decrease in Ki-67 expression. Ki-67 is an essential proliferation marker, and its expression in a tumor cell can be utilized as an indicator for prognosis, alongside other markers [33]. A reduced level of Ki-67 is characteristic of cells undergoing cell cycle arrest, which is in agreement with our results indicating higher levels of G0/G1 cell cycle arrest after treatment with compound **4** [34]. This research demonstrates that the DNMT1 inhibitor dihydroergotamine (compound **4**) modulates a multitude of tumor progression hallmarks, such as migration, apoptosis, and cell proliferation. It has previously been postulated that tumors with high intra-tumoral heterogeneity may cause patients to have poorer clinical prognosis, as it poses favorable conditions for resistance development [35]. Consequently, the plethora of different cellular pathways targeted by compound **4** may be beneficial in overcoming these resistances in highly heterogeneous tumors.

## 4. Materials and Methods

### 4.1. Cell Lines and Treatment Conditions

The drug-sensitive CCRF-CEM leukemia cell line, as well as the multidrug-resistant subline CEM/ADR5000, were kept in RPMI 1640 medium (Sigma-Aldrich, Taufkirchen, Germany) enhanced with 10% fetal bovine serum (FBS) and 1% penicillin. CEM/ADR5000 subline resistance was maintained through P-glycoprotein overexpression induced by bi-weekly treatment with 5000 nM of doxorubicin. The multidrug resistance phenotype of CEM/ADR5000 has been shown by a multitude of studies conducted by us and others [36,37,38,39]. CCRF-CEM cells and CEM/ADR5000 cells were kindly provided by Prof. Axel Sauerbrey (Department of Pediatrics, University of Jena, Germany). The adherent colon carcinoma cell line, HCT116, as well as the adherent breast cancer cell line, MDA-MB-468, were cultured in DMEM medium (Sigma-Aldrich, Taufkirchen, Germany) enhanced with 10% fetal bovine serum (FBS) and 1% penicillin/streptomycin. HCT116 cells were kindly provided by Dr. B. Vogelstein and H. Hermeking (Howard Hughes Medical Institute, Baltimore, MD, USA), and MDA-MB-468 cells were acquired from Prof. Ulrike Kämmerer, Würzburg, Germany. All cell lines were kept in a humidified incubator at 37 °C and 5% CO_2_.

### 4.2. Virtual Drug Screening

A large chemical library containing approximately 10,000 compounds from the ZINC15 clean subset, as well as 1500 FDA-approved drugs, was established. ZINC15 has an interesting feature called “tranches”, which allows the users to download the ligands based on their physical properties. The molecules in the ZINC database have different acceptance to the pan-assay interference (PAINS). Based on this PAINS (reactivity), the subsets were categorized into the following groups: (A) anodyne (no acceptance of PAINS compounds); (B) clean (allows PAINS); (C) mild (containing PAINS patterns and weakly reactive, typically as a nucleophile or electrophile); (D) reactive; and (E) unstable or irrelevant for screening.

The first database was used to identify novel compounds with epigenetic effects, and the second was used because of our interest in the drug repurposing concept. The 3D structures of the ligands were downloaded from the ZINC15 database (https://zinc15.docking.org, accessed on 27 November 2022), and the crystal structure of DNMT1 was downloaded from the Protein Data Bank (https://www.rcsb.org, accessed on 9 February 2023) (PDB code 4WXX). Initially, the ligands were energetically minimized, and the PDB file of DNMT1 was converted into a PDBQT file. In this study, in silico virtual drug screening was performed using the computational drug discovery software PyRx–Python Prescription 0.8 (https://pyrx.sourceforge.io, accessed on 3 March 2023) in order to discover new potential DNMT1 inhibitors. The screened ligands were finally ranked based on their lowest binding affinities (LBE, kcal/mol).

### 4.3. Molecular Docking

Molecular docking was performed using AutoDock 4.2.6 to further validate the binding of the top ligands revealed by virtual screening to DNMT1. All files of compound structures, as well as macromolecule structures, were converted into Protein Data Bank Partial Charge and Atom Type (PDBQT) files. While subjecting the macromolecule and ligands to molecular docking, the Lamarckian algorithm was used. Furthermore, 250 runs and 25,000,000 energy evaluations were selected, and all other docking parameters were kept as the default. The grid box was formatted to encompass the *S*-adenosylhomocysteine (SAH) of the entire protein. Docking was then executed using the services of the supercomputer MOGON at Johannes Gutenberg University (Mainz, Germany) (https://hpc.uni-mainz.de, accessed on 5 August 2023). As each investigated ligand was docked in several conformations, the conformation with the lowest binding energy was used to create a ranking of the compounds. The predicted inhibition constant (pKi), as well as the amino acid interactions, were revealed through this docking. The results were analyzed and finally visualized using MOE.

### 4.4. MOE Docking

The four best-performing compounds were selected for further molecular docking studies using Molecular Operating Environment (MOE) software version 2022.02 by the Chemical Computing Group (https://www.chemcomp.com, accessed on 4 July 2024). For the docking simulations, the X-ray crystallographic structure of human DNMT1 was extracted from the Protein Data Bank (PDB) (https://www.rcsb.org/, accessed on 4 July 2024, PDB ID 4WXX). The Amber10:EHT forcefield was assigned to the system. The structure was prepared by first removing solvent molecules. Then, the QuickPrep function was used, which protonates the system using the 3D protonate function, performs energy minimization, and repairs any missing residues. The compound’s structures were energy-minimized and washed dominantly at a pH of 7. For the docking simulation, the general docking method was chosen. The SAH binding pocket was selected as the docking site, and the protocol was configured to perform 100 initial placements of each compound. Poses were generated using the triangle matcher method and scored by London dG in MOE [40]. Fifty poses were further refined with the GBVI/WSA dG scoring function and the receptor’s induced fit mode. Three independent docking simulations were performed for each compound. Finally, the mean of the docking score (S) and standard deviation were calculated.

### 4.5. MD Simulations for Protein–Ligand Complexes

Complexes of the compounds of the docking simulations with MOE were selected as inputs for MD simulations. The pose for each compound was determined by the most favorable docking score. The system was prepared using the Amber10:EHT forcefield and underwent protonation and energy minimization using the QuickPrep function of MOE. After preparation with MOE, the files were processed utilizing NAMD 2.13 software for Win64-multicore-CUDA [41]. MOE default settings for a brief simulation of 1 ns were employed, with a timestep of 2 fs. After minimization, the system was gradually heated to 300 K over 100 ps. This was followed by an equilibration phase, first under an NVT ensemble for 100 ps at 300 K and then under an NPT ensemble for 200 ps at 300 K and 1 atm pressure. For compound **3**, the time steps were set to 1 fs. The final trajectory files were analyzed according to root mean square deviation (RMSD), root mean square fluctuation (RMSF), and the number of hydrogen bonds formed, with a cutoff of 3.5 Å during the simulation using VMD version 1.9.3 [42].

### 4.6. DNMT1 Activity Assay

The inhibition of DNMT1 activity was assessed in vitro using the DNMT1 Inhibitor Screening Assay Kit (Abcam, Cambridge, UK) in accordance with manufacturer protocol. As instructed, a 1× wash buffer was made by diluting 10× wash buffer with distilled water, and SAM was diluted with DNMT assay buffer at a 4:1 ratio. For blank wells, 27 µL of DNMT assay buffer and 3 µL of diluted SAM was added. For untreated negative control wells, 25 µL of DNMT assay buffer, 3 µL of diluted SAM, and 1 µL of purified DNMT1 enzyme (100 ng/µL) were added. Finally, for inhibitor wells, 22 µL of DNMT assay buffer, 3 µL of diluted SAM, 1 µL of DNMT1 enzyme, and 3 µL of compound (10 µM) were added. All wells were covered with Parafilm^®^ M and incubated at 37 °C for 60 min. After incubation, each well was washed three times with 150 µL of 1× wash buffer. Using the 1× wash buffer, the capture antibody was diluted at a 1:1000 ratio. Then, 50 µL of the diluted capture antibody was added to each well and incubated at RT for 60 min on an orbital shaker at 50–100 rpm. After incubation, each well was washed four times with 150 µL of 1× wash buffer. An aliquot of 50 µL of detection antibody (1:1000 dilution with 1× washing buffer) was added to each well, and the mixture was incubated again at RT for 30 min. After incubation, each well was washed with 150 µL of 1× washing buffer five times. Then, 50 µL of enhancer solution (1:5000 dilution with 1× washing buffer) was added to each well and incubated at RT for 30 min, and after incubation, each well was washed four times with 150 µL of 1× washing buffer. Afterward, 100 µL of developing solution was added to each well and incubated at RT for 5 min in the dark. Finally, 50 µL of stop solution was added to each well, and the absorbance was read on a microplate reader at 450 nm. The DNMT1 activity was calculated using the following formula:

(1) $$\mathrm{Inhibition}\;\%=1-(\frac{Inhibitor\;Sample\;OD-BLank\;OD}{No\;Inhibitor\;Control\;OD-Blank\;OD})\times \mathrm{100}\%$$

### 4.7. Resazurin Cytotoxicity Assay

The cytotoxicity of the top compounds was determined in vitro using the resazurin reduction assay following the protocol described as follows: The cell lines HCT116 and MDA-MB-468 grow adherently and were therefore seeded and incubated in 96-well plates (1 × 10^4^ cells/well) for 24 h prior to treatment, allowing attachment. The suspension cells CCRF-CEM and CEM-ADR5000 were seeded in 96-well plates (5 × 10^3^ cells/well) and immediately treated. All cells were seeded at a volume of 100 µL. The two compounds solved in DMSO were added in 10 concentrations ranging from 0.003 to 100 µM, resulting in a total volume of 200 µL/well. After incubation for 72 h, 20 µL of 0.01% resazurin solution was added to each well and again incubated. After 4 h, the Infinite^®^ M2000 Pro plate reader (Tecan, Crailsheim, Germany) was used to measure fluorescence, with an excitation wavelength of 544 nm and an emission of 590 nm [43]. In this method, the blue non-fluorescent resazurin is reduced to pink-fluorescent resorufin by metabolically active cells, allowing for a direct assessment of cell viability based on the measured fluorescence [44]. The IC_50_ values were then calculated and compared to the control cells treated with DMSO. Three repetitions of this assay were conducted, with six wells for each concentration.

### 4.8. CMV-Luc Assay in KG-1 Cells

This assay was conducted using the KG-1 cell line, which was stably transfected with the luciferase firefly gene coding for a light-emitting enzyme and cultivated in RPMI-1640 medium supplemented with 10% fetal bovine serum (FBS), 1% penicillin, and 0.5 mg/mL of geneticin. In these cells, the reporter genes were controlled by a cytomegalovirus (CMV) promoter, which is sensitive to the methylation status [45]. Cells were seeded in 96-well plates at a concentration of 20,000 cells per well and treated with three concentrations of compound **4** (10 µM, 20 µM, and 30 µM). A negative control using DMSO, as well as a positive control using the known DNMT1 inhibitor RG108, were also prepared. After 24 h of incubation, the induction of promoter activity was measured using luminescence quantification with the Infinite^®^ M2000 Pro plate reader (Tecan, Crailsheim, Germany). The data were shown in the form of fold change after treatment compared to the negative control. Three independent repetitions of the experiment were performed.

### 4.9. Migration Assay

MDA-MB-468 cells and HCT116 cells in DMEM medium (Sigma-Aldrich, Taufkirchen, Germany) enhanced with 10% fetal bovine serum (FBS) and 1% penicillin were seeded separately into 6-well plates at a density of 5 × 10^5^ cells/well and left to incubate for 24 h, after which a confluency of around 90% was achieved. The monolayer that formed was then scratched in a straight line in the middle of each well with a sterile 10 µL pipette tip. Using PBS, the wounded monolayer was washed twice to ensure debris removal. Before incubation, DMSO was added to the control wells, and to the remaining wells, fresh complete media containing 0.5 × IC_50_, 1 × IC_50_, or 2 × IC_50_ of compound **4** were added. Photographs were taken at 0 h, 24 h, and 48 h using a JuLIT™Br Live Cell Movie Analyzer (NanoEnTek Inc., Seoul, Republic of Korea) at 1× magnification and 1/3700 s of exposure time. Cell migration and wound healing were subsequently analyzed using Image J macros version 1.54g.

### 4.10. Cell Cycle

Using flow cytometry, the effect of compound **4** on cell cycle distribution was analyzed. CCRF-CEM and MDA-MB-468 cells were seeded (1 × 10^6^ cells/well) in 6-well plates and treated with compound **4** at concentrations of 0.5 × IC_50_, 1 × IC_50_, or 2 × IC_50_ or DMSO as a negative control. After 24 h of incubation, cells were centrifuged and washed with ice-cold PBS. The cell pellet was fixed using 80% ethanol and kept at −20 °C for 48 h. Then, the cells were resuspended using 1 mL PBS enhanced with 1 mg/mL RNase A (Sigma-Aldrich), as well as 50 µg/mL propidium iodide (PI) (Sigma-Aldrich) and incubated in the dark at room temperature for 15 min. The measurements were performed using a flow cytometer (BD Accuri™ C6 cytometer, BD Biosciences, San Jose, CA, USA).

### 4.11. Annexin V/PI Apoptosis Assay

To detect the apoptosis of cells treated with compound **4**, an annexin V/PI Apoptosis Detection Kit (BDbiosciences, Heidelberg, Germany) and flow cytometry (BD Accuri™ C6, BD Biosciences) were used. CCRF-CEM cells (1 × 10^6^ cells/well) were seeded into 6-well plates and treated with 0.5 × IC_50_, 1 × IC_50_, or 2 × IC_50_ of compound **4**. DMSO was applied as a negative control. After 24 h of incubation, cells were harvested and washed once with cold PBS and once with 1 × annexin binding buffer. To the cells collected through centrifugation, 5 µL of fluorochrome-conjugated annexin V was added and left at room temperature in the dark for incubation for 15 min. Finally, cells were stained using PI staining buffer (400 µL of 1 × binding buffer and 2.5 µL of PI). The results were read and analyzed using a flow cytometer and FlowJo software version 10.10.0. Experiments were repeated three times independently.

### 4.12. Fluorescence Imaging

MDA-MB-468 breast cancer cells at a density of 3 × 10^2^ cells/well were seeded in an 8-well slide chamber and incubated for 24 h. The cells were then treated with 0.5 × IC_50_, 1 × IC_50_, or 2 × IC_50_ of compound **4** and incubated again for 24 h. DMSO was used as a negative control. Subsequently, cells were covered with 4% formaldehyde diluted in 1× PBS and left to fix for 15 min at RT. The specimen was blocked for 60 min and then treated with diluted primary antibody Ki-67 (D3B5) and left to incubate overnight at 4 °C. The following day, cells were washed three times with 1× PBS to remove excess primary antibody and were then treated with diluted secondary antibody Alexa Fluor^®^ 488 Conjugate for 2 h at RT in the dark. Both primary and secondary antibodies were purchased from Cell Signaling Technology (Leiden, The Netherlands). Specimens were then washed, and DAPI was added for 5 min, followed by the addition of ibidi Mounting Medium (ibidi). Imaging was carried out using an AF7000 Widefield Fluorescence Microscope (Leica, Wetzlar, Germany) (40× magnification) at the green channel (488/510 nm) and DAPI (358/461 nm) in order to determine KI-67 localization and expression.

### 4.13. Flow Cytometry

CCRF-CEM cells were seeded in 6-well plates (1 million cells/well), treated with 0.5 × IC_50_, 1 × IC_50_, or 2 × IC_50_ of compound **4** or DMSO as a negative control, and incubated for 24 h. After centrifugation and resuspension in 100 µL of 4% formaldehyde, the cells were fixed for 15 min at RT and then washed using 1× PBS. Subsequently, the cells were permeabilized through the slow addition of ice-cold 100% methanol while carefully vortexing them. Then, they were left for 10 min on ice to permeabilize. A total of 5 × 10^5^ cells were then added to tubes and washed using centrifugation to remove excess methanol. A total of 100 µL of primary antibody Ki-67 (D3B5) was prepared in antibody dilution buffer, and then the cells were treated and incubated for 1 h at RT. After the washing steps, the cells were resuspended in 100 µL of diluted fluorochrome-conjugated secondary antibody and left to incubate for 30 min at RT in the dark. After the final washing step, the cells were resuspended in PBS, and the results were read on a flow cytometer.

### 4.14. SDS-Page and Western Blotting

CCRF-CEM cells were seeded in 6-well plates and treated with 0.5 × IC_50_, 1 × IC_50_, or 2 × IC_50_ of compound **4**, with DMSO as a negative control. Cells were incubated for 24 h and then washed with PBS. Mammalian Protein Extraction Reagent (78503, Thermo Scientific, Rockford, IL, USA) and Complete Mini protease inhibitor (Roche, Mannheim, Germany) were added at a ratio of 1:100. After carefully shaking the cells for 30 min at 4 °C, proteins were collected using centrifugation, and the protein concentration was determined using NanoDrop1000. We subsequently followed the western blot procedure, as previously described by our group [46]. In short, sodium dodecyl sulfate polyacrylamide gel electrophoresis (10% SDS-PAGE) was used to separate a 30 µL protein sample, after which the gels were transferred on a polyvinylidene fluoride (PVDF) membrane and blocked for 1 h at RT using bovine serum albumin 5% in TBST. The membranes were treated with the primary antibodies, ß-actin, PARP, caspase-3, and BCL-XL. All antibodies were purchased from Cell Signaling (Frankfurt a. M., Germany) and incubated overnight at 4 °C. The membranes were then washed with TBST 3 × 10 min, followed by the addition of the secondary antibody coupled with horseradish peroxidase (Cell Signaling Technology), and incubated for 2 h at RT. Prior to reading the results, the membranes were incubated for 3 min in the dark with Luminata Classico Western HRP substrate (Merck Millipore, Schwalbach, Germany). The visualization of the bands was performed using the Alpha Innotech FluorChem Q system (Biozym, Oldendorf, Germany), and a protein expression analysis was performed using ImageJ software version 1.54g.

### 4.15. Statistical Analysis 

All results are presented as mean values ± standard deviation (SD) of experiments performed in duplicate or triplicate. Using Student’s *t*-test, a statistical analysis was performed. The level of significance was expressed as the *p*-value < 0.05 to show the difference between treatment with compound **4** and DMSO.

## 5. Conclusions

Overall, this study elucidated the distinguished inhibitory effects of compound **4** on cell proliferation and migration, as well as the induction of late apoptosis and cell cycle arrest, all of which could be the consequence of DNMT1 inhibition. Despite the need for further in vitro and in vivo experiments to be performed on safety and the involved molecular mechanisms, compound **4** may hold high potential for future development as an anticancer drug.

## Figures and Tables

**Table 1:** Virtual screening results of the top compounds obtained from PyRx. The binding energy, molecular weight, and logP of the selected compounds are listed.

No.	ZINC ID	Pyrx Binding Energy (kcal/mol)	Molecular Weight	logP
1	ZINC04050909	−11.7	408.339	4.682
2	ZINC03360933	−11.3	487.61	4.407
3	ZINC01038993	−10.9	390.45	4.762
4	ZINC02107822	−10.9	423.516	4.416
5	ZINC03037862	−10.9	479.492	5.142
6	ZINC01038994	−10.8	394.413	4.592
7	ZINC01071494	−10.8	467.907	4.95
8	ZINC02383479	−10.8	430.508	4.962
9	ZINC02971168	−10.8	501.608	4.69
10	ZINC02731106	−10.7	484.467	3.756
11	ZINC01038992	−10.6	376.423	4.453
12	ZINC02239620	−10.6	465.509	5.937
13	ZINC02241295	−10.6	471.9	5.974
14	ZINC02347371	−10.6	469.472	5.768
15	ZINC02690584	−10.6	429.426	5.301
16	ZINC02704495	−10.6	419.383	4.522
17	ZINC02860618	−10.6	459.885	5.229
18	ZINC03668964	−10.6	519.645	7.69
19	ZINC000008220909	−12.2	665.733	0.12
20	ZINC000111460375	−11.8	562.706	4.24
21	ZINC000003978005	−11.7	583.689	2.081
22	ZINC000169289767	−11.4	872.894	6.67
23	ZINC000052955754	−11.3	581.673	1.991

**Table 2:** Molecular docking of the top selected compounds obtained from PyRx-based screening. The lowest binding energies (kcal/mol) were estimated using AutoDock 4.2.6, and the amino acids involved in the interaction with DNMT1 are listed. Amino acids in bold formed hydrogen bonds with the ligands.

	Compound	Lowest Binding Energy (kcal/mol)	pKi (nM)	Amino Acid Interactions
1	ZINC03037862-1	−10.27 ± 0.12	30.51 ± 6.25	GLN1157, MET1077, SER1076, GLY1079, PRO1080, ASN1040, LEU1594, LYS1593, LEU1590, LYS1586
2	ZINC03668964-2	−10.25 ± 0.37	36.44 ± 19.20	GLN594, VAL658, SER563, GLU562, GLU566, PRO574, ARG690, GLN687, GLN684, ARG1238, ASP571
3	ZINC02731106	−9.90 ± 0.11	56.24 ± 10.21	GLN594
4	ZINC03360933	−9.74 ± 0.37	87.12 ± 51.28	LEU1331, PHE1362, HIS1332, TRP1395, LEU1400, LYS1586, PRO1583, TYR1304, ALA1587, LEU1590, PHE1396, MET1077
5	ZINC02239620	−9.73 ± 0.46	97.42 ± 66.04	ASP569, ASP565, GLU566, ASP571, GLN687, ALA669, PRO574, GLN684, GLU572, ARG690
6	ZINC01038992	−9.71 ± 0.09	77.09 ± 12.20	GLY568, ASP569, GLN687, SER570, **ARG690**, GLU572, ASP565
7	ZINC02241295	−9.42 ± 0.20	131.36 ± 48.55	ARG1453, **PHE1492**, PRO363, GLN 1491, LEU 365, TYR 359, GLN 358, ARG1490, ALA1488
8	ZINC02347371	−9.39 ± 0.33	154.90 ± 93.95	CYS667, GLN687, ARG690, ASP571, ASP565, GLU566, GLU562, VAL658
9	ZINC01038993	−9.35 ± 0.05	141.0 ± 12.59	SER1078, PRO1080, LEU1590, **ASN1081**, PHE1362, HIS1332, LEU1331, ASN1040, TRP1395
10	ZINC01038994	−8.97 ± 0.08	266.98 ± 32.49	ALA838
11	ZINC01071494	−8.85 ± 0.10	331.18 ± 61.84	GLY1147, ASN1576, GLU1168, GLU1256, MET1169, CYS1191, ILE1167, PHE1145, ASN1267, PRO1225
12	ZINC02860618	−8.83 ± 0.02	337.46 ± 10.41	GLU1168, MET1169, GLY1223, PHE1145, PRO1225, LEU1247
13	ZINC02690584	−8.80 ± 0.22	379.17 ± 149.96	CYS1191, PHE1146, PRO1225, MET1169, GLU1168, GLY1147, ASN1578, CYS1148
14	ZINC02107822	−8.65 ± 0.41	593.29 ± 443.15	PRO363, ASP364, GLN358, GLN1491, ARG1490, PHE1492, ARG552
15	ZINC02704495	−8.54 ± 0.20	443.35 ± 276.67	GLU1168, PHE1145, GLY1147, GLY1223, SER1146, ASN1578, ASN1267, VAL1268, PRO1225
16	ZINC02971168	−8.52 ± 0.55	823.7 ± 611.19	ASP364, PRO363, THR424, PHE1492, ARG1453, ALA1488, ARG1490
17	ZINC02383479	−8.46 ± 0.25	681.75 ± 237.69	GLY568, GLU566, ASP565, GLN687, ALA669, SER570, ARG690, GLU572
18	ZINC04050909	−8.02 ± 0.005	1330 ± 16.33	VAL658, GLU566, GLU562, ASP565, PRO574, GLU572, ASP571, SER570, ARG690
	**ZINC-15 FDA**			
19	ZINC000111460375-3	−11.09 ± 0.02	7.40 ± 0.34	ARG1603, THR1602, VAL1604, LYS1323, LYS881, PRO880, ARG898
20	ZINC000003978005-4	−9.67 ± 0.22	86.65 ± 27.28	GLN358, LEU365, ASP364, PHE1492, PRO363, ARG1453, LYS505, ASN1493, ARG1490, ALA1488, MET1451
21	ZINC000052955754	−8.88 ± 0.58	437.91 ± 23.24	RG1574, TRP1170, MET1169, PHE1145, LEU1247, SER1246, PRO1225, GLY1228, PHE1229
22	ZINC000169289767	−8.72 ± 0.35	473.68 ±246.88	**ARG1310**, ASN1578, GLU1168, PRO1225, ASP521, LYS1242, ASN519, LYS1242, SER520
23	ZINC000008220909	−7.99 ± 0.03	1390.0 ± 80	SER570, GLU572, ASP571, ASP565, GLU566, VAL658, PRO574, ARG 690

**Table 3:** Docking scores and amino acid interactions of the four candidate compounds and SAH with DNMT1. Mean values and standard deviations of the docking scores were obtained from three independent docking simulations using MOE. Interacting amino acids were listed from the compound poses with the lowest docking scores. Amino acids in bold formed hydrogen bonds with the ligands.

Compound	Docking Score (kcal/mol)	Amino Acid Interactions
SAH	−8.23 ± 0.23	Phe1145, **Ser1146**, Gly1147, **Cys1148**, Gly1149, **Gly1150**, **Leu1151**, Ile1167, **Glu1168**, Met1169, Trp1170, Ala1173, Glu1189, **Asp1190**, **Cys1191**, Gly1223, Pro1225, Leu1247, **Asn1578**, Ala1579, **Val1580**
Compound **1**	−10.03 ± 0.16	Asp1143, Phe1145, Ser1146, Gly1147, Cys1148, Gly1149, Gly1150, Leu1151, Ile1167, **Glu1168**, Met1169, Glu1189, Asp1190, Cys1191, Gly1223, Pro1224, Pro1225, **Cys1226**, **Gln1227**, Leu1247, Asn1267, Val1268, Arg1310, Thr1528, Gly1577, Asn1578, Ala1579, Val1580
Compound **2**	−9.76 ± 0.15	Thr523, Thr616, **Phe1145**, Ser1146, Gly1147, Cys1148, Gly1149, Gly1150, Leu1151, Glu1168, Met1169, Asp1190, Asn1192, Gly1223, Pro1225, Gln1227, Leu1247, Thr1528, Gln1536, Arg1574, Gln1575, **Asn1578**, Ala1579, Val1580
Compound **3**	−8.08 ± 0.29	Phe1145, Glu1168, **Met1169**, Trp1170, Gly1223, Pro1224, Pro1225, Gln1227, Leu1247, **Glu1266**, Asn1267, Val1268, Arg1310, Arg1574, Asn1578, Ala1579, Val1580
Compound **4**	−9.85 ± 0.14	Thr616, **Phe1145**, Ser1146, Gly1147, Cys1148, Ile1167, **Glu1168**, Met1169, Trp1170, Ala1173, Glu1189, Asp1190, Cys1191, Asn1192, Gly1223, Pro1224, Pro1225, Cys1226, Leu1247, Glu1266, Asn1267, Val1268, Arg1574, Gln1575, Asn1578, Ala1579

**Table 4:** Cytotoxicity of compounds **2** and **4** toward leukemia, colon cancer, and breast cancer cell lines as measured by the resazurin reduction assay.

Cell Lines	IC_50_ Values of Compound 2 (µM)	IC_50_ Values of Compound 4 (µM)
CCRF-CEM	>100	18.25 ± 4.37
CEM-ADR5000	>100	>100
HCT116	>100	46.82 ± 3.04
MDA-MB-468	>100	29.42 ± 2–37

## Data Availability

All data is contained within the article and Supplementary Materials.

## Supplementary Materials

The following supporting information can be downloaded at https://www.mdpi.com/article/10.3390/ijms252211870/s1.
