# Supplementary material for: Supercomputer-Based Virtual Screening for Deoxyribonucleic Acid Methyltransferase 1 Inhibitors as Novel Anticancer Agents
Source: Int J Mol Sci. 2024 Nov 5;25(22):11870. doi: 10.3390/ijms252211870 (PMC11594074; doi:10.3390/ijms252211870)
Supplement: Supplementary file 1 [file ijms-25-11870-s001.zip › Supplementary Information.pdf]

Supplementary Information: MD Simulations

MD simulations of DNMT1 with four different compounds **1** - **4** were performed to investigate stability and flexibility profiles. To illustrate the dynamic interactions observed, a short supplementary video for each compound has been included. These videos capture the protein-compound complex trajectories.

- **Video S1**: DNMT1 with Compound **1**
- **Video S2**: DNMT1 with Compound **2**
- **Video S3**: DNMT1 with Compound **3**
- **Video S4**: DNMT1 with Compound **4**

Supplementary Materials S5–S9 present the original western blot of the cleaved PARP, B-actin, BCL-XL, and caspase-3 for CCRF-CEM cells treated with compound **4**. In addition, for caspase-3, one image with increased intensity is included to visualize the cleaved caspase-3. The first four wells represent the DMSO, 0.5 × IC_{50}, IC_{50}, and 2 × IC_{50} samples.
