# Supplementary figures and images for: Supercomputer-Based Virtual Screening for Deoxyribonucleic Acid Methyltransferase 1 Inhibitors as Novel Anticancer Agents
Source: Int J Mol Sci. 2024 Nov 5;25(22):11870. doi: 10.3390/ijms252211870 (PMC11594074; doi:10.3390/ijms252211870)

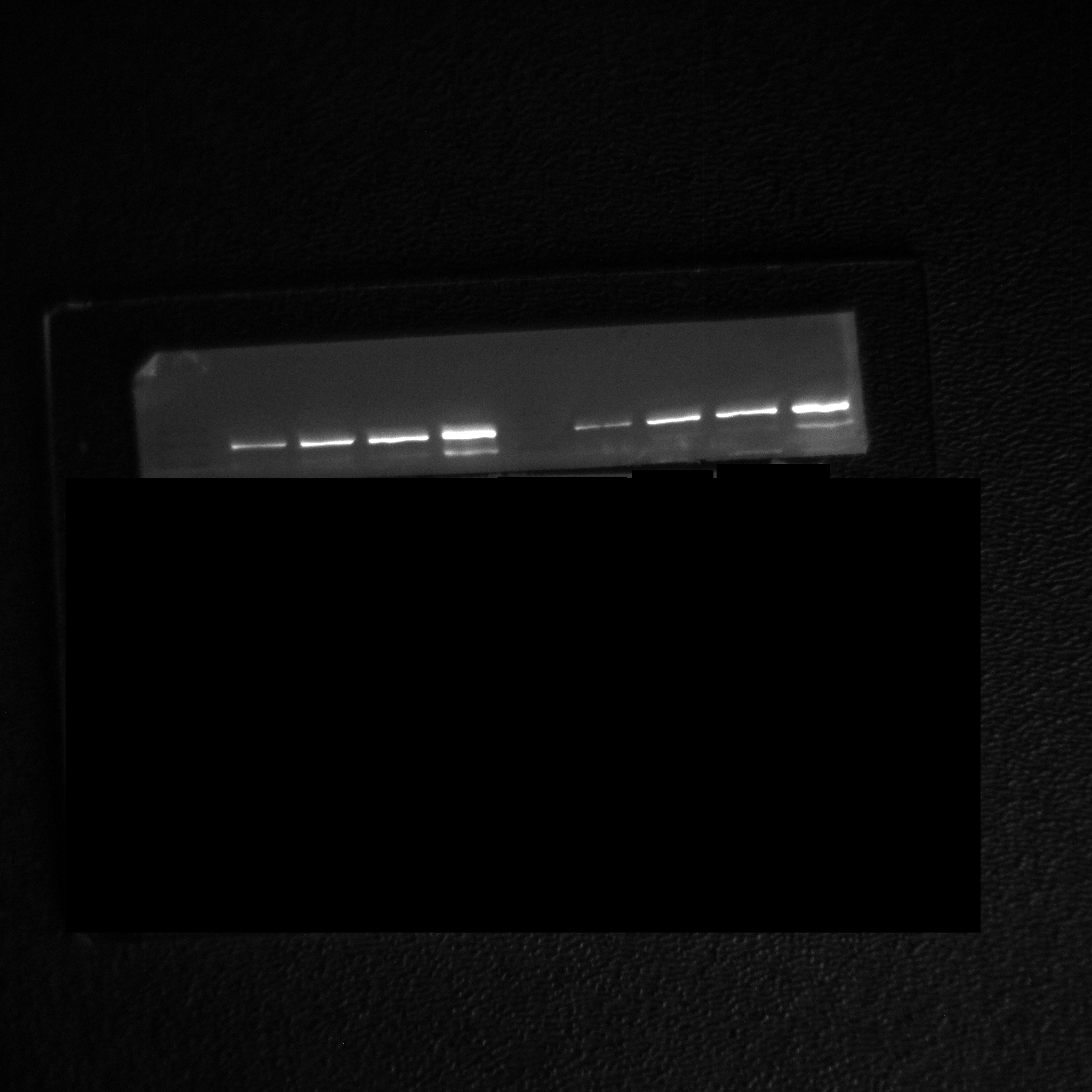

Supplement: Supplementary file 1 [file ijms-25-11870-s001.zip › S5. Parp western blot. DMSO, half ic50, ic50, 2ic50.tif]

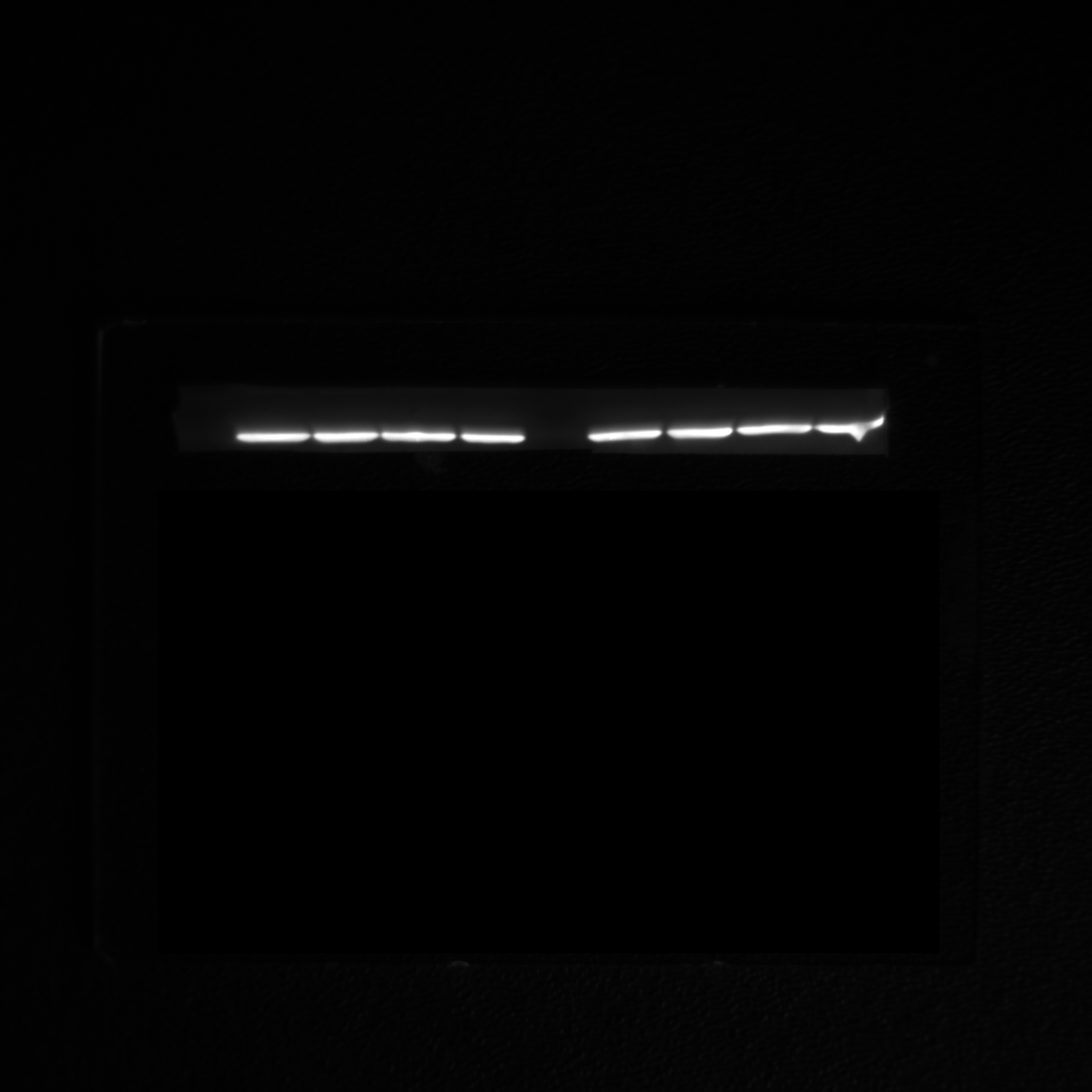

Supplement: Supplementary file 1 [file ijms-25-11870-s001.zip › S6. B-actin western blot DMSO, half ic50, ic50, 2ic50.tif]

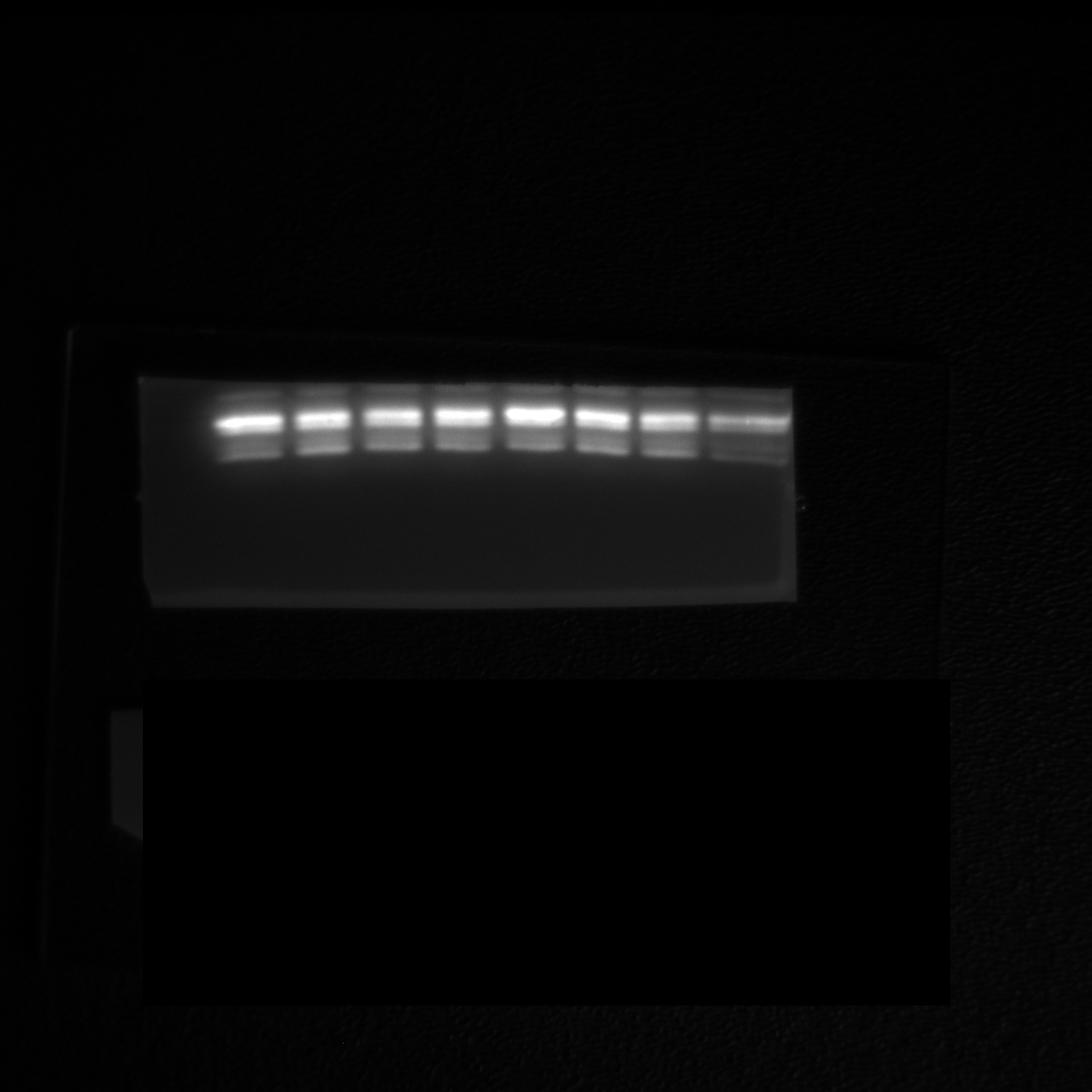

Supplement: Supplementary file 1 [file ijms-25-11870-s001.zip › S7. BCL-XL western blot, DMSO, half ic50, ic50, 2ic50.tif]

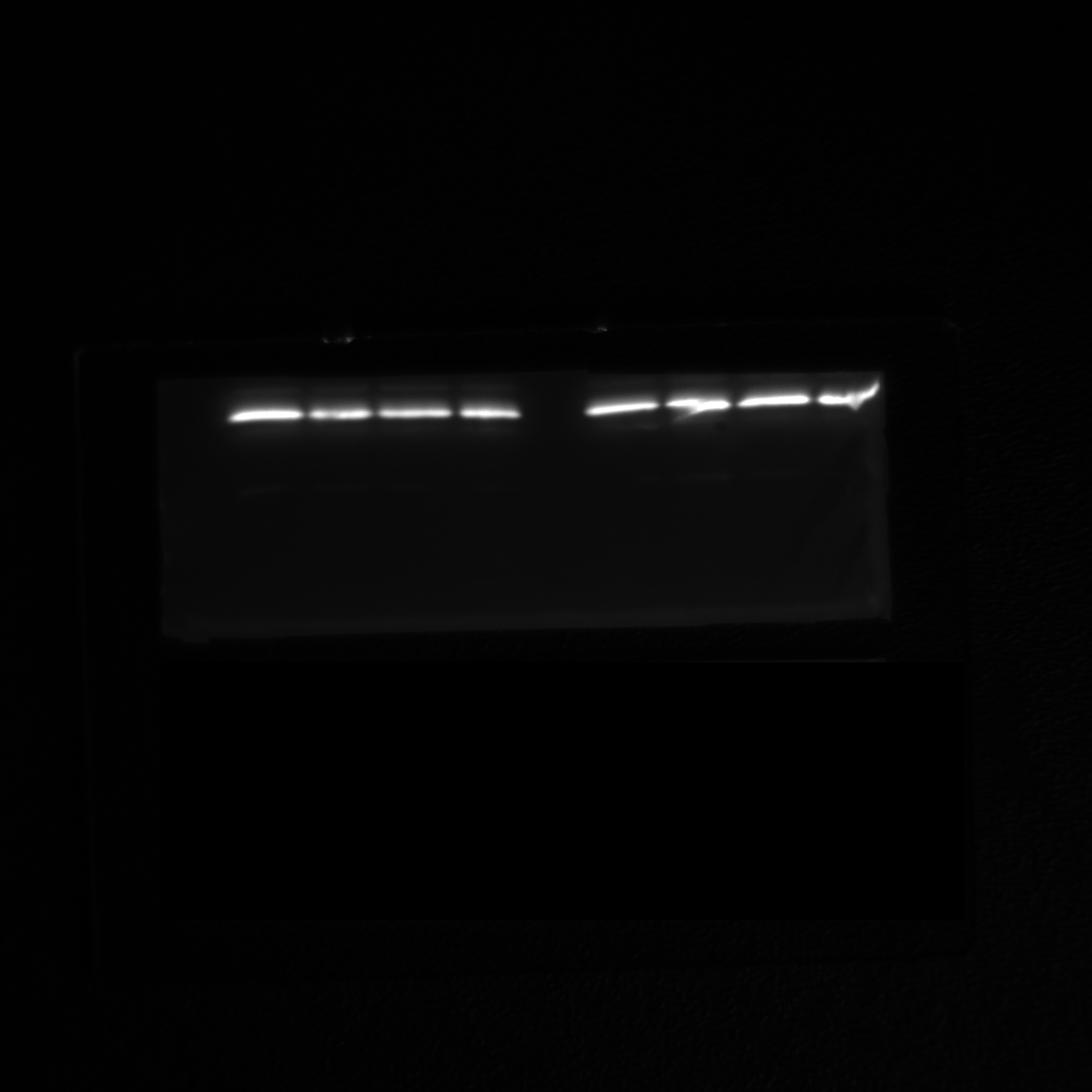

Supplement: Supplementary file 1 [file ijms-25-11870-s001.zip › S8. caspase 3 western blot, DMSO, half, ic50, 2ic50 .tif]

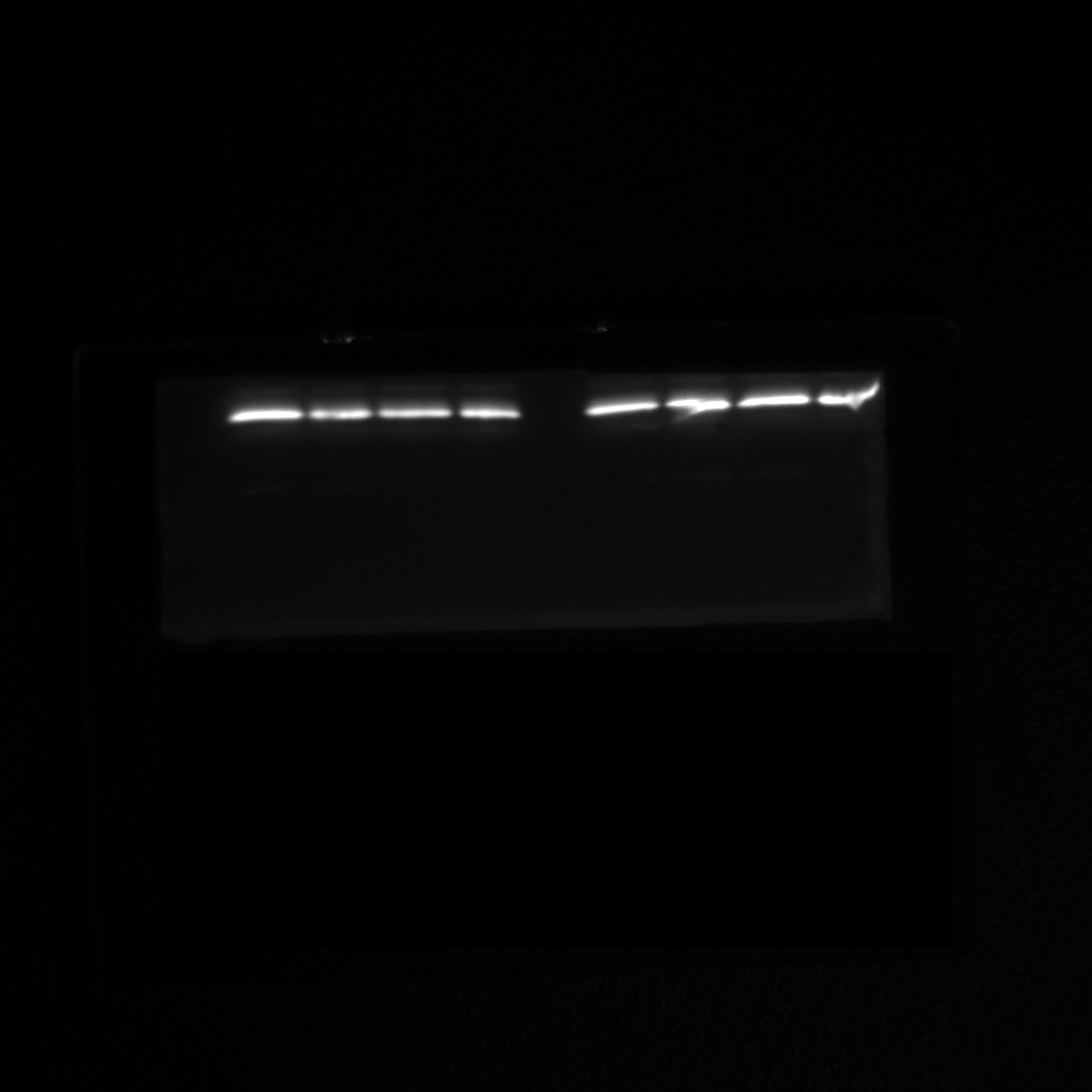

Supplement: Supplementary file 1 [file ijms-25-11870-s001.zip › S9. caspase 3 increase intensity western blot, DMSO, half, ic50, 2ic50 .tif]
